# Vitrectomy with internal limiting membrane peeling versus nonsurgical treatment for diabetic macular edema with massive hard exudates

**DOI:** 10.1371/journal.pone.0236867

**Published:** 2020-07-31

**Authors:** Hsuan-Chieh Lin, Chung-May Yang, San-Ni Chen, Yi-Ting Hsieh

**Affiliations:** 1 Department of Ophthalmology, National Taiwan University Hospital, Taipei, Taiwan; 2 Department of Ophthalmology, National Taiwan University Hospital, Hsinchu branch, Hsinchu, Taiwan; 3 School of Medicine, National Taiwan University, Taipei, Taiwan; 4 Department of Ophthalmology, Changhua Christian Hospital, Changhua, Taiwan; 5 School of Medicine, Chung Shan Medical University, Taichung, Taiwan; 6 Department of Optometry, College of Nursing and Health Sciences, Da-Yeh University, Changhua, Taiwan; National Yang-Ming University Hospital, TAIWAN

## Abstract

**Purpose:**

To compare the anatomical and functional outcomes of severe diabetic macular edema (DME) with massive hard exudates managed by pars plana vitrectomy (PPV) with internal limiting membrane (ILM) peeling or nonsurgical treatment.

**Methods:**

We retrospectively reviewed 40 eyes with DME and massive hard exudates treated with either PPV with ILM peeling (vitrectomy group, 21 eyes) or nonsurgical treatment with anti-vascular endothelium growth factor (VEGF) and/or steroids (nonsurgical group, 19 eyes). Changes in best-corrected visual acuity (BCVA) and central retinal thickness (CRT) and resolution of macular hard exudates were compared between the two groups.

**Results:**

After treatment, CRT decreased steadily in the vitrectomy group but fluctuated in the nonsurgical group. Compared with eyes in the nonsurgical group, eyes in the vitrectomy group had better visual improvement (*P* < 0.05 at 6 and 12 months and the final visit) and greater decrease in CRT (*P* < 0.05 at 3 and 6 months and the final visit) after adjustment for baseline BCVA. Hard exudates resolved more rapidly in the vitrectomy group than in the nonsurgical group, with 94.1% versus 47.4% eyes showing significant absorption after 6 months of the treatment (*P* = 0.003). In the vitrectomy group, 62% eyes did not require any further injections for treating DME after the operation.

**Conclusions:**

PPV with ILM peeling resulted in rapid resolution of hard exudates with significant anatomical and functional improvement in DME with massive hard exudates.

## Introduction

Diabetic macular edema (DME) is a major cause of vision loss in patients with diabetic retinopathy [1, 2]. Several treatment modalities such as focal laser photocoagulation [3], intravitreal or subtenon injection of triamcinolone [4, 5], sustained-release corticosteroids implant [6, 7], and intravitreal injection (IVI) of anti-vascular endothelial growth factor (VEGF) [8, 9] have been proposed to manage DME. Currently, IVIs of anti-VEGF are considered the first-line treatment of choice for DME, whereas IVI of corticosteroids may be considered for pseudophakic eyes or patients with a high risk of thromboembolic events. Laser treatment continues to play an important role in preventing moderate vision loss in non-center-involving DME. Surgical interventions are normally reserved for those with evidence of vitreoretinal traction [10, 11]. Studies have demonstrated that vitrectomy has long-term benefits for diffuse DME [12–14], even for those without a thickened and taut posterior hyaloid [15–17]. In addition, vitrectomy combined with internal limiting membrane (ILM) peeling has shown favorable anatomical and functional outcomes [18–22].

DME with massive hard exudates is a severe form of DME; it usually indicates excessive leakage and is considered a poor visual prognostic factor [23]. Hard exudates are lipids and proteinaceous materials that deposit within the neurosensory retina and in the subretinal space. Massive hard exudates tend to deposit in the foveal area and form fibrotic plaques, causing damage to photoreceptors with irreversible central visual loss. Although medical and surgical treatments have been proposed for enhancing the resolution of exudates [24–32], the disease remains a major challenge. Our previous report demonstrated that pars plana vitrectomy (PPV) with posterior hyaloid removal, focal macular endolaser, and panretinal photocoagulation (PRP) can reduce massive macular exudates [33]. In addition, ILM peeling can remove the tangential traction exerted by the residual cortical vitreous and ILM, prevent postoperative epiretinal membrane (ERM) formation, and improve oxygen supply by removing the diffusion barrier [34, 35]. Thus, we hypothesized that PPV combined with ILM peeling may facilitate the resolution of exudates and represent a favorable treatment for DME with massive hard exudates. Therefore, this study aimed to evaluate the one-year anatomical and functional outcomes of DME with massive hard exudates managed by PPV with ILM peeling and compare them with those managed by nonsurgical treatments.

## Materials and methods

### Study population

This was a bicenter, retrospective, consecutive case series of patients with severe DME and massive hard exudates treated at National Taiwan University Hospital or Changhua Christian Hospital from October 2009 to September 2015. Inclusion criteria were as follows: (1) presence of DME with massive hard exudates, which were defined as fovea-involved, single or multiple patches of confluent hard exudates with a total area of >3 disc areas at the posterior pole confirmed by color fundus photography; (2) presence of intraretinal and/or subretinal hyperreflective materials involving the fovea confirmed by optical coherence topography (OCT); and (3) a best-corrected visual acuity (BCVA) of ≤20/200. Eyes with previous vitreoretinal surgery, evidence of a taut posterior hyaloid or vitreomacular traction determined by OCT images, vitreous hemorrhage, active fibrovascular proliferation, other retinal vascular diseases, choroidal neovascularization, or a follow-up period of <12 months were excluded from the study.

Eyes fulfilling the aforementioned criteria were divided into two groups: the study group, in which all eyes underwent PPV with ILM peeling, and the control group, in which all eyes underwent only nonsurgical treatments. Eyes treated with only vitrectomy and no ILM peeling were excluded from the study. In this bicenter study, patients treated by three ophthalmologists (CM Yang, SN Chen, and YT Hsieh) were retrospectively enrolled. CM Yang treated all his patients with DME and massive hard exudates using vitrectomy and ILM peeling during Oct 2009 and Sep 2015. SN Chen and YT Hsieh treated all their patients with DME and massive hard exudates using medical treatment with anti-VEGF during Oct 2009 and Jan 2013, and treated such patients using vitrectomy and ILM peeling during Feb 2013 and Sep 2015. In the study group, eyes that had received anti-VEGF, steroid or laser treatment within three months of operation were excluded. In the control group, all eyes were treatment-naïve. For patients with concurrent DME with massive hard exudates in both eyes, the more severe one received surgical treatment, and the less severe one received nonsurgical treatment. This study adhered to the tenets of the Declaration of Helsinki and was approved by the Institutional Review Boards of the National Taiwan University Hospital and Changhua Christian Hospital.

### Treatment protocols

#### Study group: Vitrectomy with ILM peeling

All eyes in the study group underwent similar surgical procedures. Briefly, a standard 23-gauge vitrectomy system was set up. After core vitrectomy, triamcinolone acetonide was injected to identify and facilitate the removal of the posterior hyaloid if no posterior vitreous detachment (PVD) was observed. After complete vitrectomy, indocyanine green (ICG; 25 mg ICG in 15 mL of 5% glucose) was slowly injected above the macula. Excess ICG was immediately washed out and the ILM staining pattern was observed. Homogeneous staining indicated no ERM, whereas scattered staining or poor staining indicated the presence of ERM and/or residual vitreous remnant. ILM peeling was initiated at the site of positive ICG staining. The ILM and overlying ERM were peeled off the retinal surface in a circumferential manner for at least 3.5-disc diameters centered at the fovea. After mild focal laser was applied to extrafoveal microaneurysms within the arcade, peripheral scatter laser was applied to the equator and slightly beyond as complete PRP. Bevacizumab (1.25 mg) was injected into the vitreous cavity at the end of surgery. All operations were performed by one of the three experienced surgeons (C-MY, S-NC, and Y-TH) applying similar surgical principles and techniques. During the postoperative follow-up period, IVI of anti-VEGF (bevacizumab 1.25 mg or ranibizumab 0.5 mg) was administered as required. In addition, IVI of triamcinolone acetonide (IVTA) at 4 mg/0.05 mL per dose or posterior subtenon injection of triamcinolone acetonide (PSTA) at 40 mg/0.5 mL per dose was administered as supplementary treatment if required.

#### Control group: Nonsurgical treatments only

In the control group, all patients were administered two or three consecutive IVIs of bevacizumab 1.25 mg or ranibizumab 0.5 mg every month as the loading treatment. After the loading phase, supplementary treatments were provided following similar protocols as in the vitrectomy group. In principle, IVI of anti-VEGF was administered as required. IVTA or PSTA was added to treatment if macular thickening persistently or recurrently developed. Focal laser was applied to the leaking microaneurysms when macular thickness returned to within the normal range. Anti-VEGF treatment was resumed if persistent or recurrent macular edema causing central macular thickness greater than 300 μm was detected by OCT after the aforementioned treatment regimen.

### Outcome measurements

Each patient underwent complete clinical ophthalmic examinations including BCVA assessment using the Snellen chart, slit-lamp examination, dilated fundus examination, color fundus photography, fluorescein angiography, and OCT (Cirrus OCT; Carl Zeiss Meditec, Dublin, CA, USA) at baseline. BCVA was converted into the logarithm of the minimal angle of resolution (logMAR) for analysis. Counting fingers at 50 cm was converted to 2.0 and hand motion was converted to 3.0 in logMAR [36]. BCVA, color fundus photography, and OCT were re-examined at 3, 6, and 12 months postoperatively and as required. Hard exudate areas were measured on the fundus photographs using ImageJ version 1.50 (National Institutes of Health, Bethesda, MD, USA) by one assessor (HC Lin) who was masked for the study group. Central retinal thickness (CRT) measured by OCT was recorded. Medical history, ophthalmic history, severity of diabetic retinopathy, and treatment-related complications were collected and recorded in detail. In addition, intraoperative findings such as the presence of PVD and ERM/vitreous remnant and the size of ILM peeling area were recorded. The main outcome measurements for data analysis were post-treatment BCVA changes, CRT changes, and resolution status of hard exudate at 3, 6, and 12 months postoperatively and at the final visit. Significant absorption of hard exudates was defined as >50% decrease in the area of hard exudates from baseline, and complete resolution of hard exudates was defined as no visible hard exudates within the area confined by the disc, arcade, and temporal margin of 5-disc diameters from the disc [3].

### Statistical analysis

The Mann–Whitney U test was performed to compare the post-treatment BCVA and CRT changes between the vitrectomy and control groups. The Fisher’s exact test was used to compare the proportions of eyes with significant/complete resolution of hard exudates between the two groups. Multiple linear mixed models were used to identify the significant factors associated with post-treatment BCVA and CRT changes with the subject as a random effect to adjust for correlations between the two eyes of the same individual. The candidate factors were age, sex, vitrectomy group versus control group, and cataract operation from baseline to the study time point. A *P* value of <0.05 was considered statistically significant. All statistical analyses were conducted using SPSS version 17.0 (SPSS Inc., Chicago, IL, USA).

## Results

This study recruited 40 eyes of 34 patients (23 men and 11 women), with 21 eyes in the vitrectomy group and 19 eyes in the control group. Both eyes of six patients were recruited for study and among them, both eyes of three patients were treated with nonsurgical treatments only. For the other three patients, the baseline BCVA was similar in their both eyes and only the eyes with larger areas of hard exudates received vitrectomy. The mean age of the patients was 58.5 ± 7.7 years (range: 42–78 years). All patients had a follow-up period of at least 12 months, and the mean follow-up period was 20.2 ± 8.3 months (range: 12–38 months). No significant differences were noted between the two groups at baseline for BCVA, CRT, hard exudate area, percentages of proliferative diabetic retinopathy, duration of diabetes, or any associated underlying diseases such as hypertension, dyslipidemia, anemia, and chronic kidney disease. The basic characteristics of patients of both the groups are summarized in Table 1.

**Table 1:** Demographic and baseline data in the vitrectomy group and the control group.

	Vitrectomy group (n = 21)	Control group (n = 19)	*P* value
**Age (year)**	60.1 ± 7.9	56.8 ± 7.2	0.19
**Male (%)**	67%	74%	0.64
**Follow-up duration (month)**	21.4 ± 8.7	18.8 ± 7.8	0.3
**Baseline LogMAR**	1.65 ± 0.38	1.46 ± 0.34	0.09
**Baseline CRT (μm)**	481 ± 196	491 ± 133	0.85
**Baseline hard exudate area (disc area)**	4.1 ± 2.1	4.2 ± 1.4	0.84
**DM duration (year)**	11.7 ± 6.9	10.7 ± 7.9	0.73
**PDR (%)**	71%	78%	0.58
**HTN (%)**	76%	63%	0.37
**Dyslipidemia (%)**	10%	15%	0.57
**CKD (%)**	19%	31%	0.36
**Anemia (%)**	24%	36%	0.37

CKD, chronic kidney disease; CRT, central macular thickness; DM, diabetes mellitus; HTN, hypertension; LogMAR, logarithm of the minimal angle of resolution; PDR, proliferative diabetic retinopathy; PPV, pars plana vitrectomy; SD, standard deviation.

### Intraoperative findings

During the operation in the vitrectomy group, it was found that 14 (67%) eyes had complete PVD and 7 (33%) eyes had incomplete PVD or no PVD. Non-homogenous ICG staining of the macula before ILM peeling, which indicated the presence of ERM/vitreous remnant, was noted in 16 (76%) eyes.

### Visual improvement and associated factors

**Fig 1:**
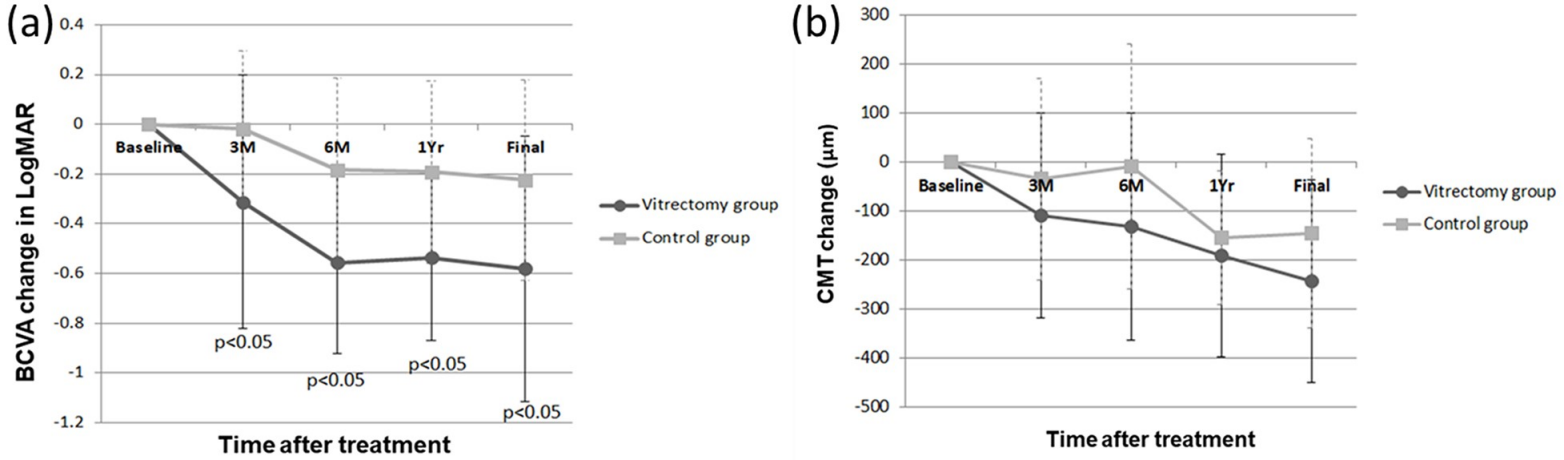
(a) Best-corrected visual acuity (BCVA) changes and (b) central macular thickness (CRT) changes after treatment in the vitrectomy group and the control group. (a) This figure shows changes of best-corrected visual acuity (BCVA) in logarithm of the minimal angle of resolution (LogMAR; mean ± standard deviation) in the vitrectomy group (circle) and the control group (square). The improvement of visual acuity was significantly better in the vitrectomy group at every time point (*P* = 0.035 at 3 months, *P* = 0.003 at 6 months, *P* = 0.003 at 1 year, and *P* = 0.024 at final visit). (b) This figure shows changes of central macular thickness (CRT) (μm; mean ± standard deviation) in the vitrectomy group (circle) and the control group (square). The CRT of the vitrectomy group decreased steadily during the follow-up period, whereas the CRT of the control group fluctuated and repeated treatments were needed. There was no significant difference in the change of CRT between the vitrectomy group and control group.

The mean logMAR of BCVA at baseline was 1.65 ± 0.38 in the vitrectomy group and 1.46 ± 0.34 in the control group. No significant difference was noted between the two groups (*P* = 0.087). After treatment, the mean BCVA significantly improved in both the groups compared with that at baseline (*P* < 0.05 for all postoperative time points in the vitrectomy group and for the time points at 6 months or later in the control group). The final BCVA (in logMAR) was 1.07 ± 0.66 in the vitrectomy group and 1.23 ± 0.44 in the control group (*P* = 0.40). Visual improvement detected by change in logMAR was found to be significantly better in the vitrectomy group than in the control group at 3 months (−0.31 ± 0.51 versus −0.02 ± 0.31, *P* = 0.035), 6 months (−0.56 ± 0.37 versus −0.18 ± 0.37, *P* = 0.003), 12 months (−0.54 ± 0.33 versus −0.19 ± 0.37, *P* = 0.003), and the final visit (−0.58 ± 0.53 versus −0.22 ± 0.40, *P* = 0.024; Fig 1A).

Multiple linear regression analysis was performed to identify the significant factors associated with post-treatment BCVA change. After adjusting for baseline BCVA and other baseline characteristics, eyes treated with PPV and ILM peeling had better visual improvement than those treated with nonsurgical treatments at 6 months (*P* = 0.020), 12 months, (*P* = 0.029), and the final visit (*P* = 0.012). In addition, younger patients tended to have better final visual improvement (*P* = 0.001; Table 2A).

**Table 2:** Multiple linear regression analysis for post-treatment BCVA change and post-treatment CRT change.

**(a)**	**Change in BCVA**							
	**3 months**		**6 months**		**12 months**		**Final**	
Factors	Coefficient	*P* value	Coefficient	*P* value	Coefficient	*P* value	Coefficient	*P* value
**PPV**	-0.167	0.290	-0.329	0.020	-0.283	0.029	-0.409	0.012
**Age**	-0.004	0.660	0.008	0.326	0.008	0.282	0.032	0.001
**Gender**	0.002	0.991	0.017	0.902	-0.023	0.859	-0.135	0.380
**Baseline BCVA**	-0.241	0.242	-0.257	0.152	-0.198	0.222	-0.084	0.669
**Cataract op**	-0.466	0.107	-0.109	0.659	-0.258	0.206	-0.146	0.422
**(b)**	**Change in CRT**							
	**3 months**		**6 months**		**12 months**		**Final**	
Factors	Coefficient	*P* value	Coefficient	*P* value	Coefficient	*P* value	Coefficient	*P* value
**PPV**	-110.617	0.064	-178.622	0.013	-69.143	0.074	-111.711	0.014
**Age**	6.726	0.105	4.425	0.351	3.160	0.203	2.592	0.374
**Gender**	-17.124	0.774	-2.537	0.973	32.732	0.398	43.078	0.336
**Baseline CRT**	-0.884	<0.001	-0.906	<0.001	-0.905	<0.001	-1.018	<0.001
**Cataract op**	95.103	0.354	293.489	0.056	86.076	0.169	-4.852	0.930

BCVA: best-corrected visual acuity, CRT: central retinal thickness, PPV: pars plana vitrectomy, op: operation.

### Anatomical improvement and associated factors

The mean baseline CRT was similar in both the groups (481 ± 196 μm in the vitrectomy group and 491 ± 133 μm in the control group, *P* = 0.85). Fig 1B shows that the mean CRT in the vitrectomy group decreased steadily during the follow-up period (change in CRT after surgery: at 3 months = −109 ± 209 μm, at 6 months = −132 ± 232 μm, at 12 months = −191 ± 206 μm, and at the final visit = −243 ± 206 μm). However, the mean CRT in the control group fluctuated and decreased more slowly (change in CRT after treatments: at 3 months = −34 ± 206 μm, at 6 months = −9 ± 250 μm, at 12 months = −155 ± 136 μm, and at the final visit = −146 ± 194 μm) and required repeated treatments. No significant difference was observed in CRT change between the vitrectomy and control groups (*P* > 0.05 for all time points). However, the final CRT was significantly thinner in the vitrectomy group than in the control group (238 ± 44 μm versus 346 ± 166 μm, *P* = 0.007).

Multiple linear regression analysis was performed to identify the significant factors associated with post-treatment CRT change. After adjusting for baseline CRT and other baseline characteristics, decrease in CRT was greater in eyes treated with PPV and ILM peeling than in those treated with nonsurgical treatments at 6 months (*P* = 0.013) and the final visit (*P* = 0.014). No other factors were found to be significantly associated with CRT change (Table 2B).

### Resolution of hard exudates

**Fig 2:**
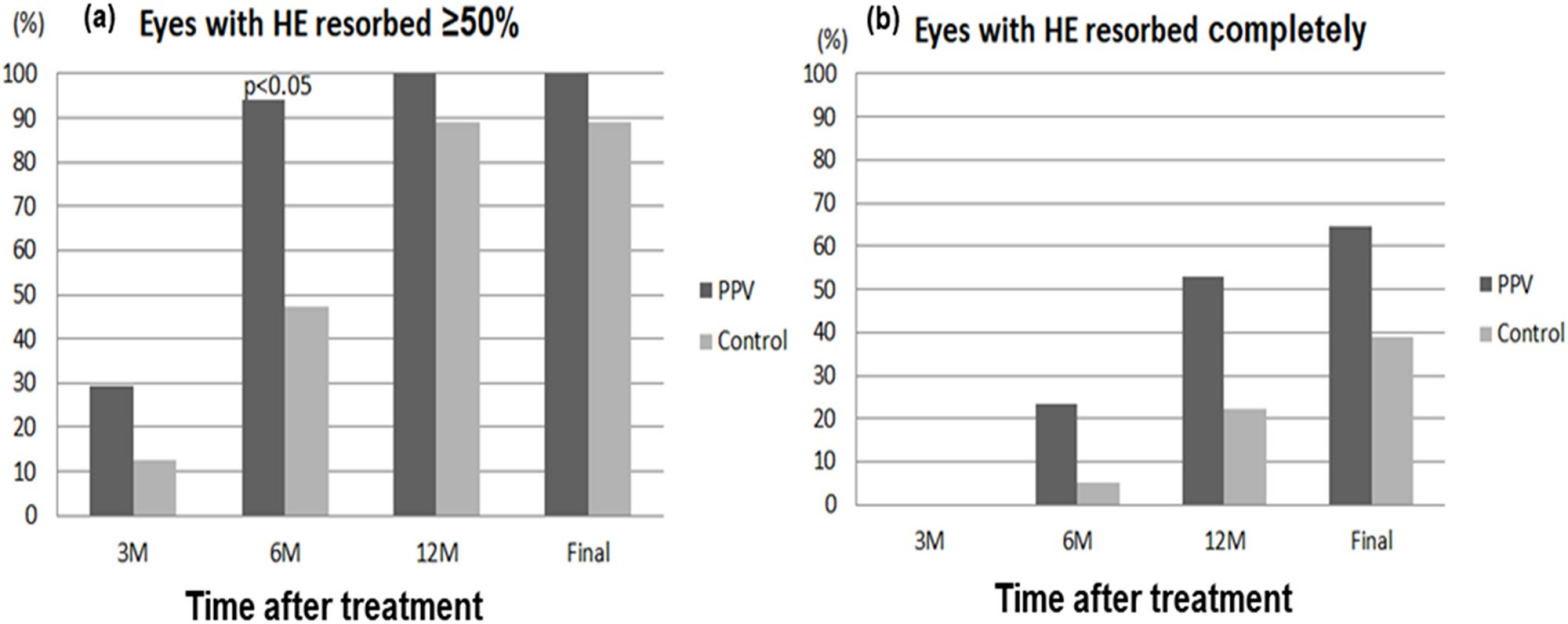
The proportion of eyes with (a) significant and (b) complete resolution of hard exudates. (a) The proportion of eyes with significant absorption of the hard exudates was 29.41%, 94.12%, 100%, and 100% in the vitrectomy group and 12.5%, 47.37%, 88.89% and 88.89% in the control group at post-treatment 3 months, 6 months, 12 months, and final visit. (Fisher exact test, *P* = 0.0034 at post-treatment 6 months). (b) Complete resolution of hard exudates at 3, 6, 12 months after treatment and final visit was found in 0%, 23.53%, 52.94%, and 64.71% of eyes in the vitrectomy group and 0%, 5.26%, 22.22%, and 38.88% in the control group.

**Fig 3:**
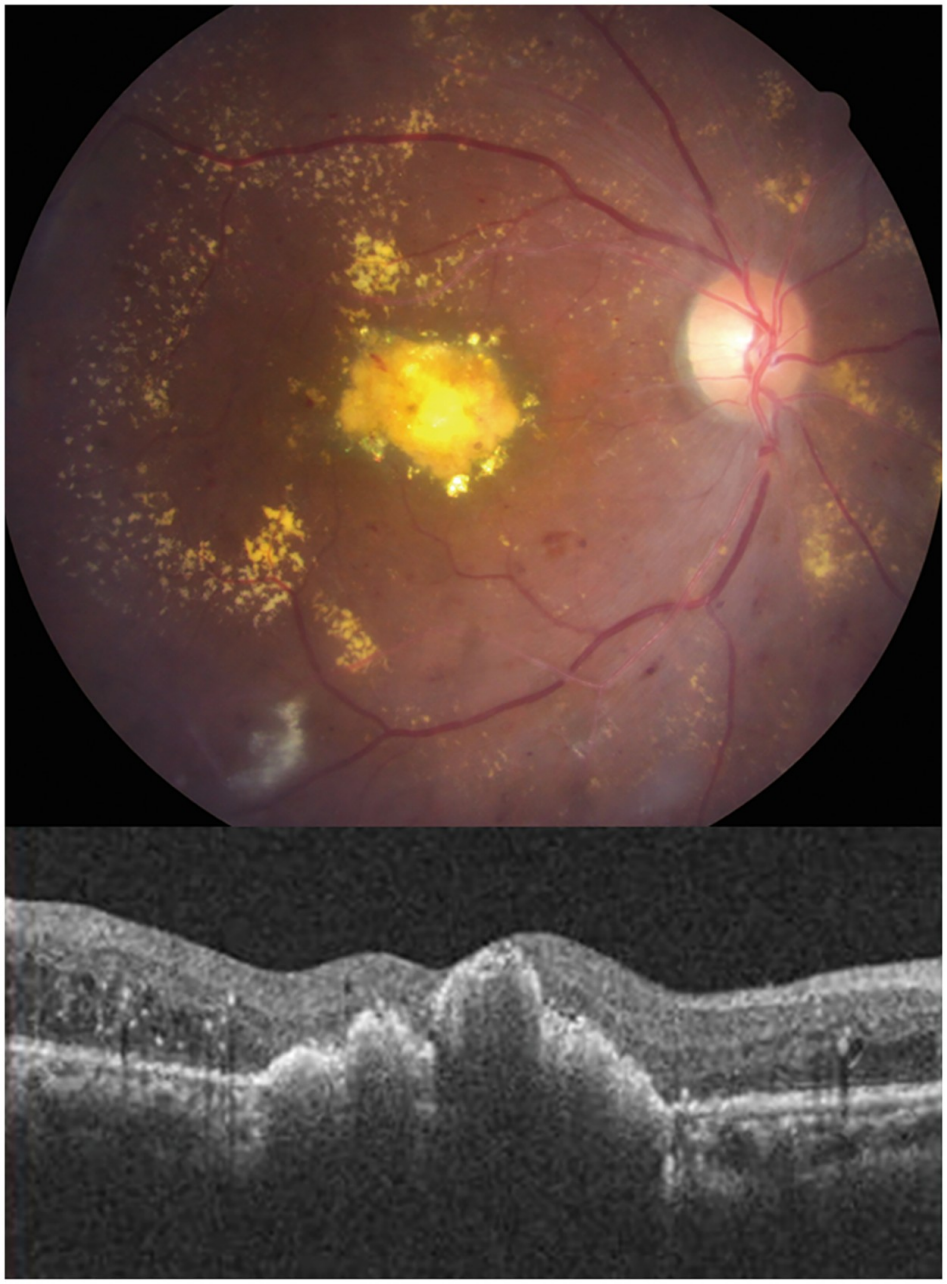
Subfoveal plaque formation after treatment of diabetic macular edema with massive hard exudates. A 62-year-old male with type 2 diabetes mellitus and hypertension received pars plana vitrectomy with internal limiting membrane peeling for diabetic macular edema with massive hard exudates in the right eye. Six months postoperatively, subfoveal plaque formation was found. Visual acuity was 20/200.

**Fig 4:**
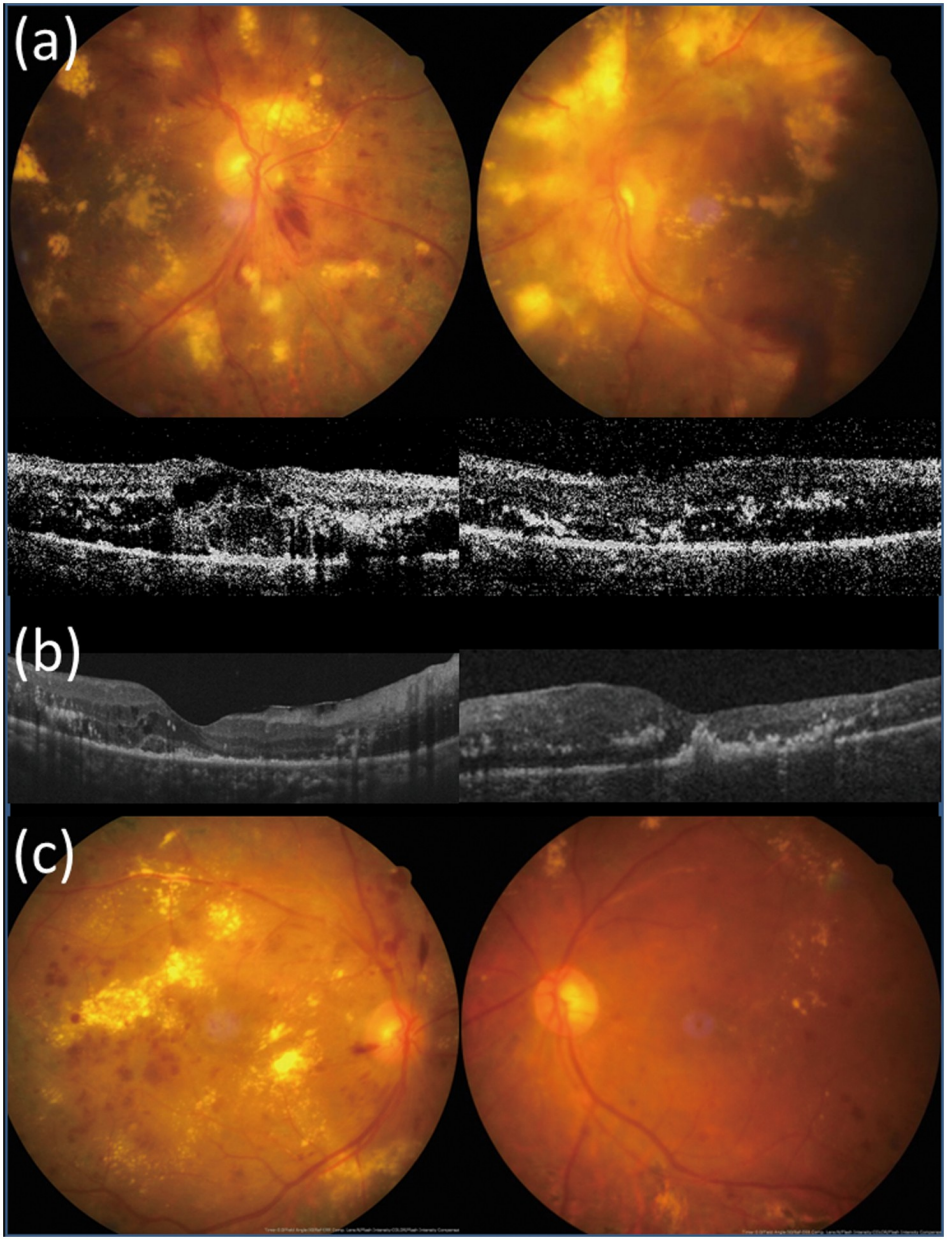
A patient with bilateral DME and massive hard exudates receiving different treatment strategies in both eyes. (a) A 63-year-old female with hypertension and type 2 diabetes mellitus for 20 years presented with bilateral diabetic macular edema with massive hard exudates, unresponsive to intravitreal bevacizumab injection and macular photocoagulation. Pars plana vitrectomy with internal limiting membrane peeling and focal laser were performed in the left eye. Pre-operative visual acuity (VA) was counting fingers in the left eye and central macular thickness (CRT) was 396 μm. VA in the right eye was 20/200 and CRT was 380 μm at the same time. (b) One month post-operatively, CRT decreased to 296 μm in the left eye. (c) Eight months post-operatively, hard exudates resorbed significantly and VA improved to 20/125 in the left eye. VA of the right eye remained poor (20/400) due to persistent hard exudates despite repeated intravitreal bevacizumab injections, posterior sub-Tenon injections of triamcinolone, and macular laser photocoagulation.

At baseline, the mean areas of hard exudate in the vitrectomy and control groups were 4.1 ± 2.1 and 4.2 ± 1.4-disc areas, respectively (*P* = 0.732). The hard exudates were reabsorbed more rapidly in the vitrectomy group than in the control group (Fig 2). After 6 months of surgery, significant absorption of hard exudates was observed in 94% of eyes in the vitrectomy group compared with only 47% of eyes in the control group (*P* = 0.003). At 12 months after surgery, complete resolution of hard exudates was noted in 53% and 22% of eyes in the vitrectomy and control groups, respectively (*P* = 0.085). Subfoveal plaque or nodule formation after treatment was observed in 33% (5/15) of eyes in the vitrectomy group and 37% (7/19) of eyes in the control group (Fig 3). In the vitrectomy group, the final BCVA was significantly better among those without than among those with subretinal hyperreflective plaque at the fovea (LogMAR 0.73 ± 0.37 versus 1.76 ± 0.89, *P* = 0.014). However, in the control group, no significant difference was noted in the final BCVA between those without and with subfoveal plaque (LogMAR 1.12 ± 0.46 versus 1.40 ± 0.32, *P* = 0.16). Subretinal plaque formation was not significantly associated with the treatment method (surgery versus nonsurgical treatment), baseline CRT, and hard exudate area. Representative images of a patient with bilateral DME and massive hard exudates treated with different treatment strategies are presented in Fig 4.

### Anti-VEGF and steroid use

The mean numbers of total IVIs of anti-VEGF, IVTA, and PSTA administered during the first year were 1.76 ± 1.41 in the vitrectomy group (including the intravitreal bevacizumab after the end of the surgery) and 3.56 ± 2.31 in the control group (*P* < 0.001). In the vitrectomy group, 13 (62%) eyes did not receive any further injections within one year after the operation, 4 (19%) eyes received only one injection, and another 4 (19%) eyes received two or more injections within 1 year of the operation.

### Complications

No serious adverse events developed in either group. The intraocular pressures were within the controllable range in all eyes. One eye in the vitrectomy group developed a lamellar macular hole after 5 months of the surgery. Two eyes in the control group developed non-clearing vitreous hemorrhage after 10 months of the initial treatment and required surgical treatment. During the follow-up period, 50% (4/8) of the phakic eyes in the vitrectomy group and 7.14% (1/14) in the control group developed significant cataract. All these eyes underwent cataract operation with good visual recovery.

## Discussion

Many studies have demonstrated the efficacy of IVI of anti-VEGF in treating DME. However, several reasons supporting surgical intervention are available. For instance, studies have indicated that diabetic eyes have a thickened ILM compared with non-diabetic eyes that may cause structural and functional disturbance of water movement between the vitreous and retina [37]. In addition, the posterior hyaloid is thickened and abundant in cells such as fibrocytes, macrophages, and myofibroblast-like cells [38]. The hyalocytes can transform into myofibroblasts, making the posterior hyaloid to become contractile [39, 40]. Moreover, the vitreous in diabetic eyes contains VEGF and other cytokines that may increase intraocular inflammation. Anti-angiogenic shift in the vitreous was observed after vitrectomy in eyes with proliferative diabetic retinopathy (PDR) [41]. All the aforementioned changes are important factors in inducing macular thickening. Hence, some researchers consider pathologic diabetic eyes to be “diabetic vitreoretinopathy [42].” PPV can address these pathological processes in a single setting.

Retinal hard exudates are a result of lipoprotein extravasation and its subsequent deposition in the extracellular space secondary to the breakdown of the blood-retinal barrier. Massive hard exudates tend to be associated with PDR. In the present study, 75% of the eyes had PDR. There are two possible reasons for this close association. First, eyes with PDR usually have extensive non-perfusion areas and produce excessive VEGF, which in turn greatly increases vascular permeability and causes out-pouring and accumulation of hard exudates. Second, eyes with PDR are frequently treated with PRP. However, PRP can induce inflammation and VEGF surge resulting in severe macular edema with massive hard exudates [43]. Severe hard exudates have been recognized to be the strongest risk factor for the development of subretinal plaque or fibrosis and have been associated with poor visual outcomes [23, 44].

Few studies have specifically investigated the treatment for massive hard exudates [24–29, 31, 32]. Subretinal removal of massive submacular hard exudates has been shown to improve visual acuity [24, 31]. However, visual improvement did not show significant differences compared to those that only received PPV. This was probably due to the irreversible retinal atrophy and degenerative changes in the affected eyes [32]. Macular hole, retinal break, submacular hemorrhage, and neurosensory retina degeneration may develop after subretinal removal of hard exudates [24, 31, 32]. Our previous study [33] found that vitrectomy with removal of the attached posterior hyaloid and focal posterior and peripheral scatter laser may dramatically reduce macular edema and exudates. However, the study was performed when OCT was not available, and it failed to address eyes that already had PVD. The evolution of hard exudates may not always be in parallel with change in macular thickness and macular cysts. Thus, monitoring hard exudates and macular edema with OCT can provide more information regarding the evolution of macular pathological changes, which was not performed in our previous study. In addition, the use of anti-VEGF as the standard treatment for DME had not been established at the time of our previous study. Therefore, comparing the evolution of hard exudates, macular edema, and visual results between surgical and medical treatments based on OCT remains a clinically relevant and important concern. Several OCT biomarkers such as inner segment-outer segment (IS-OS) layer, external limiting membrane layer, choroidal thickness, submacular fluid, hyper-reflective foci, and disorganization of retinal inner layers have been identified for DME treated with anti-VEGF, dexamethasone implant, or vitrectomy [45–48]. It is difficult to analyze the morphology and thickness of the choroid using spectral-domain OCT (SD-OCT), which was used in the present study, compared with enhance depth imaging SD-OCT or swept-source OCT. However, OCT angiography may be useful in detecting macular ischemia in cases of DME with massive hard exudates [49].

In the present study, many cases had tightly adherent ERM with or without PVD. ILM peeling assured complete removal of ERM, thus further decreasing preretinal traction. We specifically investigated visual acuity improvement, decrease in macular thickness, and reduction in the size of hard exudates in the vitrectomy and control groups. Visual improvement was significantly better in the vitrectomy group than in the control group at each time point. Similar trend was observed for CRT changes, without statistical difference between the groups. CRT decreased markedly within 3 months and remained stable afterwards in the vitrectomy group. By contrast, in the control group, CRT fluctuated during the follow-up period, which is likely the result of the temporary effects of conventional treatments, and repeated treatments were required. The time duration for the macula to return to normal thickness was longer and final CRT was significantly thicker in the control group than in the vitrectomy group. Although not statistically significant, a trend toward poorer baseline visual acuity was observed in the vitrectomy group. After adjusting for baseline BCVA, multiple regression analysis revealed that eyes treated with PPV and ILM peeling had better visual improvement than those treated with nonsurgical treatments. In addition, younger age was associated with greater visual improvement at the final visit. Thicker baseline CRT was associated with more reduction in CRT after treatment, and eyes treated with PPV and ILM peeling had more improvement in CRT at 6-month post-treatment and final visits. These results are consistent with other reports focused on DME [23, 50, 51]. Furthermore, a trend of more rapid resorption of hard exudates was observed in the vitrectomy group (Fig 2), which might explain the greater visual improvement after PPV.

**Fig 5:**
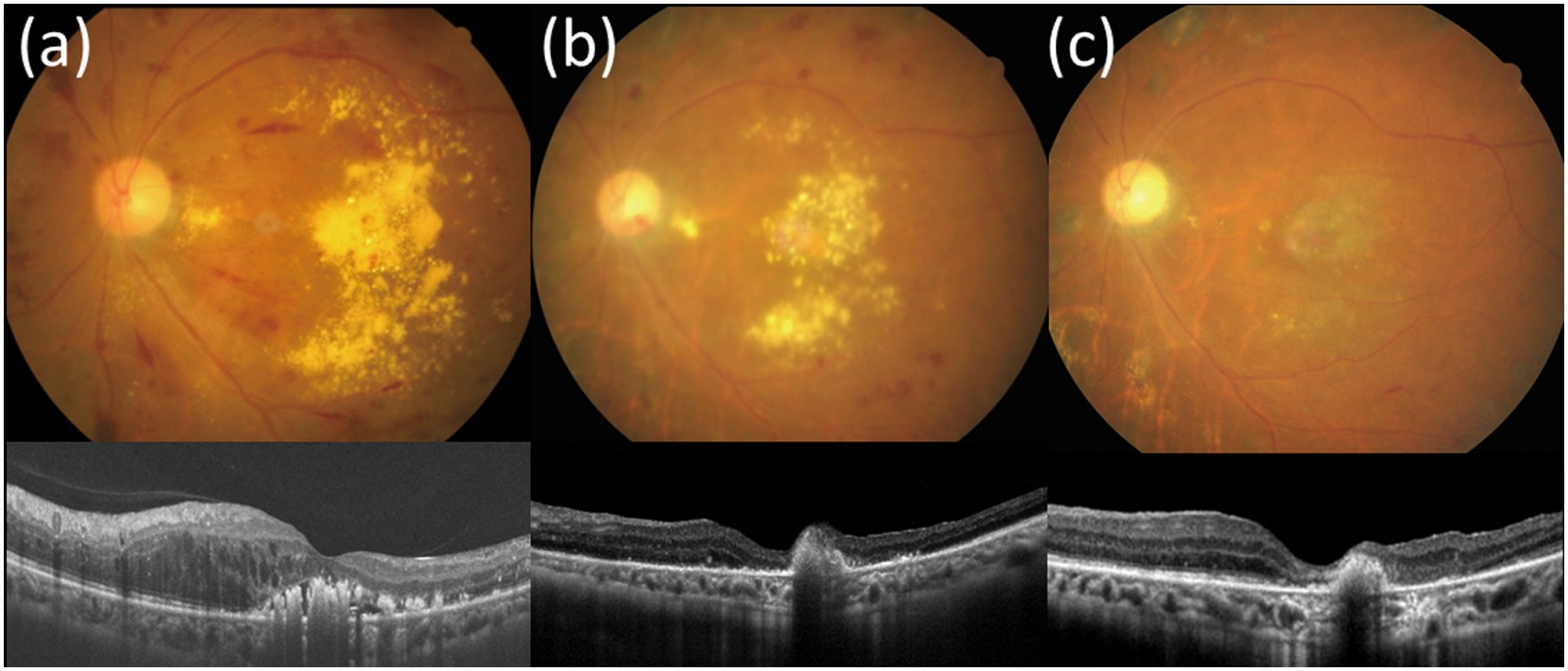
Macular atrophy after longstanding diabetic macular edema with massive hard exudates. (a) A 47-year-old male with type 2 diabetes mellitus, hypertension, chronic kidney disease, and anemia suffered from diabetic macular edema in both eyes with massive hard exudates in the left eye. IVI of bevacizumab, posterior sub-Tenon injection of triamcinolone, and macular photocoagulation were applied, but the response was poor. Pars plana vitrectomy with internal membrane peeling and PRP were performed on the left eye. Pre-operative visual acuity (VA) was counting fingers and central macular thickness (CRT) was 302 μm in the left eye. (b) Three months later, a subretinal plaque was observed in the left eye and VA remained counting fingers. CRT was 178 μm in the left eye. (c) One year postoperatively, massive hard exudates reabsorbed completely in the left eye. VA was 20/400 due to atrophic changes in the macula.

Elevated levels of serum lipid are associated with an increased risk of development of retinal hard exudates [52]. In the present study, patients of both the groups had similar underlying diseases such as hypertension, dyslipidemia, anemia, and chronic kidney disease associated with the disease. Moreover, all cases with dyslipidemia were controlled with medication. Hard exudates were completely reabsorbed within 1 year in 52.9% of eyes in the vitrectomy group, and subfoveal plaques or nodule formation was considered a major factor impairing visual recovery. The percentage of subfoveal plaques or nodule formation was similar in both the groups. Hard exudates are originally lipids and proteinaceous materials that are effluxed from the retinal vessels to the outer plexiform layer. However, it is believed that in eyes with massive hard exudates, accumulation of deposits are usually longstanding and may extend to the subretinal region [31], thereby causing irreversible degenerative or atrophic changes (Fig 5). Focal laser treatment performed during vitrectomy and as supplementary treatment in the control group might reduce the leakage from microaneurysms and possibly aid in the resolution of hard exudates. Vitrectomy could promote the resolution of hard exudates by improving oxygenation through removal of mediators, which resulted in increased vascular permeability, and ILM peeling further eliminated the barrier to cytokines and oxygen [53]. Iglicki et al. described a novel OCT finding of outer retinal hyper-reflective deposits in DME eyes appearing after PPV with ILM peeling and disappearing over the postoperative course, which indicates a result of sudden desinflammation shortly after the surgery with gradual resolution enabled by improved oxygenation [54]. The final BCVA was significantly better among those without than among those with subfoveal plaques/nodule formation in the vitrectomy group, indicating that DME should be managed promptly before massive hard exudates deposit in the macula. The lack of significantly better visual acuity among cases without than among cases with subfoveal plaques in the control group may be partially explained by the overall poor vision in this group.

No serious complication was noted in either group. Only one eye that developed a lamellar hole after vitrectomy had an extremely thick macula (1017 μm) with schisis changes preoperatively and the posterior hyaloid was not detached from the macula. No cases of endophthalmitis or uncontrollable intraocular pressure occurred. Therefore, PPV with ILM peeling is a relatively safe procedure with careful case selection. However, despite macular thickness normalization, two cases in the control group developed vitreous hemorrhage resulting in severe visual loss. In these two cases, vitrectomy was performed to regain vision. This type of downturn in the clinical course indicates a high risk of neovascularization in cases with massive hard exudates, even under anti-VEGF treatment. This observation further justifies the application of scatter laser during surgery for such cases.

This study has a few limitations such as the retrospective design, lack of randomization, and a small number of cases. In addition, our control group did not follow the standard protocol of anti-VEGF treatment guideline strictly. Several factors contributed to the less than optimal number of IVIs of anti-VEGF in the real-world setting, including patient-related factors such as financial concerns, transport issues, poor motivation, or inconvenience due to other diabetes-related comorbidities. During the study period, no insurance reimbursement for anti-VEGF treatment for DME was available in Taiwan; hence, patients usually received a pro re nata protocol. In fact, the mean injection number of 3.6 within 1 year was not quite low compared with other published real-world studies [55, 56]. Although the use of combination medical treatment prevents adequate assessment of the effect of individual therapy, the treatment regimen in this study likely reflects the real-world condition and clearly demonstrated favorable functional and anatomical outcomes as well as reduction of treatment burden in the surgery group. The results of our case-control study suggest that PPV with ILM peeling may eliminate the inflammatory cytokines in the vitreous [41], remove the tangential traction [39, 40] exerted by the residual cortical vitreous and ILM, prevent ERM formation, and may be a favorable alternative treatment option for DME with massive hard exudates. Future prospective and randomized studies are warranted to refine current algorithm for DME management. Ophthalmologists who still prefer intravitreal anti-VEGF or steroid implant first, we suggest switching to vitrectomy with ILM peeling if no improvement is noted after 3 months.

## Conclusions

PPV combined with ILM peeling may result in rapid resolution of hard exudates as well as provide long-term anatomical and functional benefits, avoiding possible complications related to multiple IVIs and huge treatment burden. Thus, PPV combined with ILM peeling may be a favorable alternative treatment option for DME with massive hard exudates.

## Supporting information

S1 File Data set. (XLS)

S2 File Certificate for English editing. (PDF)
