# Supplementary material for: Vitrectomy with internal limiting membrane peeling versus nonsurgical treatment for diabetic macular edema with massive hard exudates
Source: PLoS One. 2020 Jul 31;15(7):e0236867. doi: 10.1371/journal.pone.0236867 (PMC7394381; doi:10.1371/journal.pone.0236867)
Supplement: S2 File — (PDF) [file pone.0236867.s002.pdf]

CERTIFICATE OF ENGLISH EDITING

This document certifies that the paper listed below has been edited to ensure that the language is clear and free of errors. The edit was performed by professional editors at Editage, a division of Cactus Communications.The intent of the author's message was not altered in any way during the editing process. The quality of the edit has been guaranteed, with the assumption that our suggested changes have been accepted and have not been further altered without the knowledge of our editors.

Title Of The Paper

Vitrectomy with Internal Limiting Membrane Peeling versus Nonsurgical Treatment for Diabetic Macular Edema with Massive Hard Exudates

Authors

Hsuan-Chieh Lin, Chung-May Yang, San-Ni Chen, Yi-Ting Hsieh

Job Code

CLXZL_1

Signature

Vikas Narang

Vikas Narang,
Chief Operating Officer,
Editage

Date of Issue
June 19, 2020

Editage, a brand of Cactus Communications, offers professional English language editing and publication support services to authors engaged in over 500 areas of research. Through its community of experienced editors, which includes doctors, engineers, published scientists, and researchers with peer review experience, Editage has successfully helped authors get published in internationally reputed journals. Authors who work with Editage are guaranteed excellent language quality and timely delivery.

INFORMATION SECURITY MANAGEMENT SYSTEM DNV·GL ISO/IEC 27001

CACTUS

Contact Editage

Worldwide	Japan	Korea	China	Brazil	Taiwan
request@editage.com	submissions@editage.com	submit-korea@editage.com	fabiao@editage.cn	contato@editage.com	submitjobs@editage.com
+1 877-334-8243	+81 03-6868-3348	1544-9241	400-005-6055	0800-892-20-97	02 2657 0306
www.editage.com	www.editage.jp	www.editage.co.kr	www.editage.cn	www.editage.com.br	www.editage.com.tw
